# Bcl-xL Reduces Chinese Giant Salamander Iridovirus-Induced Mitochondrial Apoptosis by Interacting with Bak and Inhibiting the p53 Pathway

**DOI:** 10.3390/v13112224

**Published:** 2021-11-04

**Authors:** Yiqun Li, Yuding Fan, Yong Zhou, Nan Jiang, Mingyang Xue, Yan Meng, Wenzhi Liu, Jingjing Zhang, Ge Lin, Lingbing Zeng

**Affiliations:** 1 Yangtze River Fisheries Research Institute, Chinese Academy of Fishery Sciences, Wuhan 430223, China; liyq@yfi.ac.cn (Y.L.); fanyd@yfi.ac.cn (Y.F.); zhouy@yfi.ac.cn (Y.Z.); n851027@yfi.ac.cn (N.J.); xmy@yfi.ac.cn (M.X.); mengy@yfi.ac.cn (Y.M.); liuwenzhialisa@yfi.ac.cn (W.L.); 18838285282@163.com (J.Z.); linge0310@163.com (G.L.); 2 National Demonstration Center for Experimental Fisheries Science Education, Shanghai Ocean University, Shanghai 201306, China

**Keywords:** Bcl-xL, chinese giant salamander iridovirus, mitochondria, apoptosis, Bak, p53

## Abstract

Chinese giant salamander iridovirus (GSIV) infection could lead to mitochondrial apoptosis in this animal, a process that involves B-cell lymphoma-2 (BCL-2) superfamily molecules. The mRNA expression level of Bcl-xL, a crucial antiapoptotic molecule in the BCL-2 family, was reduced in early infection and increased in late infection. However, the molecular mechanism remains unknown. In this study, the function and regulatory mechanisms of Chinese giant salamander (*Andrias davidianus*) Bcl-xL (AdBcl-xL) during GSIV infection were investigated. Western blotting assays revealed that the level of Bcl-xL protein was downregulated markedly as the infection progressed. Plasmids expressing *AdBcl-xL* or *AdBcl-xL* short interfering RNAs were separately constructed and transfected into Chinese giant salamander muscle cells. Confocal microscopy showed that overexpressed AdBcl-xL was translocated to the mitochondria after infection with GSIV. Additionally, flow cytometry analysis demonstrated that apoptotic progress was reduced in both *AdBcl-xL*-overexpressing cells compared with those in the control, while apoptotic progress was enhanced in cells silenced for *AdBcl-xL*. A lower number of copies of virus major capsid protein genes and a reduced protein synthesis were confirmed in *AdBcl-xL*-overexpressing cells. Moreover, AdBcl-xL could bind directly to the proapoptotic molecule AdBak with or without GSIV infection. In addition, the p53 level was inhibited and the mRNA expression levels of crucial regulatory molecules in the p53 pathway were regulated in *AdBcl-xL*-overexpressing cells during GSIV infection. These results suggest that AdBcl-xL plays negative roles in GSIV-induced mitochondrial apoptosis and virus replication by binding to AdBak and inhibiting p53 activation.

## 1. Introduction

The B-cell lymphoma-2 (BCL-2) superfamily, which comprises both proapoptotic molecules and antiapoptotic molecules, are proteins characterized by the existence of short conserved sequence regions (the BCL-2 homology (BH) motifs), playing crucial roles in determining whether the cells survive or die [1,2]. The ratio of antagonist (antiapoptotic) to agonist (proapoptotic) molecules dictates whether a cell responds to a proximal apoptotic stimulus [3]. Bcl-xL is an antiapoptotic molecule and shares conserved BH motifs and a C-terminal transmembrane (TM) region [4,5]. In mammals, when cells suffer apoptotic stimulus, Bak (BCL2 antagonist/killer 1) and Bax (BCL2-associated X, apoptosis regulator) are activated and then oligomerize on the mitochondrial outer membrane (MOM) to permeabilize the MOM by forming pores [6]. Bcl-xL is present on the MOM to prevent the oligomerization of proapoptotic molecules (Bax/Bak) and control the balance of the mitochondrial membrane potential (MMP) [7]. Inactivation or downregulation of antiapoptotic BCL-2 proteins results in Bak autoactivation, and physiological restraint of Bak by Bcl-xL has been proven to be essential for cell survival [8,9]. The loss of MMP can lead to the activation of caspase family members (including caspase-3 and caspase-9), which initiate apoptosis [10]. It has been reported that p53 can activate the mitochondrial pathway and regulate Bcl-2 proteins [11].

Previous studies showed that the BCL-2 superfamily plays a functional role during virus infection. Members of the BCL-2 superfamily were found to regulate the cell cycle during virus infection, while some of them are recruited by the virus for their replication and release. Overexpression of Bcl-xL inhibited the activation of multiple caspases and virus release following coxsackievirus B3 infection [12]. HIV and Hepatitis B virus proteins might modulate the levels of BCL-2 superfamily proteins through independent interactions for virus replication and to induce cell death [13,14]. In humans, Bcl-2 acts to restrict replication and delays virus-induced programmed cell death during the early stage of Semliki Forest virus infection [15]. In addition to RNA viruses, DNA viruses have evolved a capacity to control cell death [16]. For example, adenovirus, Kaposi’s sarcoma-associated herpesvirus (KSHV), Epstein-Barr virus (EBV), and murine gammaherpesvirus 68 (HV68) encode homologs of mammalian BCL2: ADE1B19K, KSHV vBCL2, BHRF-1, and HV68 M11, respectively [17]. EBV encodes a microRNA, miR-BART5, that targets BH3-only proteins, including the p53 upregulated modulator of apoptosis (PUMA) [18]. Giant sea perch iridovirus infection upregulates Bak expression and leads to apoptotic death of fish cells [19]. Bcl-2 and Bcl-xL overexpression can overwhelm the function of the proapoptotic protein Bax during GSIV-ST kinase induction of Bax expression and block iridovirus serine/threonine kinase-induced mitochondrial cell death in GF-1 cells [20]. However, in amphibians, the functional roles of antiapoptotic BCL-2 family members remain unclear.

Chinese giant salamander iridovirus (GSIV) belongs to a large dsDNA virus family, Iridoviridae, which displays icosahedral symmetry, with a diameter of approximately 120–200 nm [21]. GSIV is the main viral pathogen of Chinese giant salamander (Andrias davidianus) and leads to severe disease of this species in aquaculture [22]. To date, no effective prevention and treatment methods are available for GSIV infection. In our previous study, GSIV infection triggered typical apoptotic cell death through both the extrinsic and intrinsic pathways [23]. In addition, the protein levels of p53 increased during GSIV-induced apoptosis. Moreover, Bid (BH3-interacting domain death agonist), a proapoptotic BH3-only protein of the BCL-2 superfamily, played a positive role during GSIV-induced apoptosis [24]. Furthermore, the expression level of Bcl-xL mRNA was decreased during early infection and then upregulated during late infection. However, the functions of these antiapoptotic proteins during GSIV infection remain unknown. Further investigation is required to determine the molecular mechanism of antiapoptotic proteins of the BCL-2 superfamily in GSIV-induced apoptosis.

In the present study, the characteristics and function of an antiapoptotic protein (Bcl-xL) of the BCL-2 superfamily in Chinese giant salamanders were investigated. The results demonstrate that the protein can suppress GSIV-induced apoptosis and inhibit virus replication. Moreover, it directly interacts with Bak and suppresses the activation of the p53 pathway during GSIV infection, which increases our understanding of the regulatory mechanisms that are active during iridovirus infection.

## 2. Materials and Methods

### 2.1. Virus and Cells

The giant salamander iridovirus (GSIV) was isolated and identified from diseased Chinese giant salamanders in our laboratory [22]. The Chinese giant salamander muscle cell line (GSM cells) was generously donated by Prof. Qiya Zhang (Institute of Hydrobiology, Chinese Academy of Sciences, Wuhan, China) and grown in Medium 199 (Hyclone, Logan, UT, USA) supplemented with 10% fetal bovine serum (FBS) at 20 °C.

### 2.2. RNA Isolation, cDNA Synthesis and Gene Cloning

GSM cells were collected and used for total RNA extraction with an EZNA Total RNA Kit (Omega Bio-Tek, Winooski, VT, USA). The RNA was treated with RNase-free DNase I (Takara, Dalian, China). The quality of the RNA was examined by determining the 260/280 absorbance ratio using a Nano Drop One spectrophotometer (Thermo Scientific, Waltham, MA, USA) and by gel electrophoresis. Reverse transcription to cDNA was carried out according to the protocol of the RevertAid First Strand cDNA Synthesis Kit (Thermo Scientific). The open reading frames (ORFs) of AdBcl-xL and AdBak were first determined using de novo transcriptome sequencing of Chinese giant salamanders [25]. The ORFs of AdBcl-xL and AdBak were amplified using PCR with specific primers (P1, P2, P3, and P4; Table 1), inserted into the pMD19-T simple vector (Takara, Shiga, Japan), and sequenced using sequencing primers.

### 2.3. Sequence Analysis

Sequence alignment was performed using CLUSTALW (https://www.genome.jp/tools-bin/clustalw, accessed on 2 February 2021), and images were generated using ESPript 3.0 (http://espript.ibcp.fr/ESPript/cgi-bin/ESPript.cgi, accessed on 2 February 2021). The phylogenetic analysis was performed using the neighbor-joining algorithm of MEGA 6.0 [26]. Motifs were predicted using Multiple Em for Motif Elicitation (MEME, https://meme-suite.org/meme/, accessed on 22 January 2021). The accession numbers of selected Bcl-xL proteins from the National Center for Biotechnology Information Reference Sequence database (http://www.ncbi.nlm.nih.gov/RefSeq/, accessed on 2 February 2021) are as follows: Mus musculus Bcl-xL, AAA51039.1; Homo sapiens Bcl- xL, CAA80661.1; Lonchura striata Bcl-xL, AAY42379.1; Xenopus laevis Bcl-xL, AAI10791.1; Danio rerio Bcl-xL, NP_571882.1; Ctenopharyngodon idella Bcl-xL, AXF53966.1; Clarias magur Bcl-xL, KAF5903480.1; Xenopus tropicalis Bcl-xL, BAB62748.1; Lithobates catesbeianus Bcl-xL, ACO51639.1; Trachemys scripta elegans Bcl-xL, ACQ42286.1; Epinephelus coioides Bcl-xL, AYA72661.1; Gadus morhua Bcl-xL, ACZ62647.1.

### 2.4. Plasmids Construction and Transfection

The pET-30a, pCDNA3.1-flag, pCDNA3.1-Myc-HisA, pEGFP-N1, and pDsRed-Monomer-N1 plasmids were purchased from Miaolingbio (Wuhan, China). The ORFs of AdBcl-xL and AdBak were amplified using primers with NheI/HindIII, EcoRI-BamHI, and XhoI-BamHI cleavage site sequences added to the 5′ end, respectively. The PCR products of AdBcl-xL were inserted into pET-30a, pCDNA3.1-flag, and pDsRed-Monomer-N1, respectively. The PCR products of AdBak were inserted into pCDNA3.1-Myc-HisA and pEGFP-N1.

Recombinant plasmids pCDNA3.1-flag-Bcl-xL, pDsRed-Monomer-N1-Bcl-xL, pCDNA3.1-Myc-HisA-Bak, and pEGFP-N1-Bak from transformed Escherichia coli cells were extracted using an Endo-Free Plasmid Midi Kit (Omega Bio-Tek). Expression constructs and the empty vectors were then introduced into GSM cells. Briefly, GSM cells were seeded into 6-well plates at a density of 2 × 106 cells/mL and cultured in medium M199 containing 10% FBS (Gibco, Grand Island, NY, USA). After 24 h, the cells were transfected with a mixture containing 4 µg of plasmid and 10 µL of Lipofectamine™ 2000 (Invitrogen, Waltham, MA, USA) in 500 µL of M199 medium per well, according to the manufacturer’s instructions. The medium was changed after 6 h, the cells were washed with phosphate-buffered saline (PBS), and then cultured in fresh M199 containing 10% FBS. At 48 h post transfection, the cells were harvested. Recombinant protein expression from pCDNA3.1-flag-Bcl-xL and pCDNA3.1-Myc-HisA-Bak was detected by Western blotting using mouse anti-Flag or anti-Myc antibodies, while recombinant protein expression from pDsRed-Monomer-N1-Bcl-xL and pEGFP-N1-Bak was examined using fluorescence microscopy (Olympus, Tokyo, Japan) (Figure S4).

### 2.5. Western Blot

The recombinant plasmid pET-30a-AdBcl-xL was transformed separately into Escherichia coli Transetta (DE3) (Takara, Japan). After propagation of positive transformants, Isopropyl β-d-1-thiogalactopyranoside induction, recombinant protein purification, and quantification of their concentrations, the purified rAdBcl-xL was immunized separately into rabbits four times to acquire polyclonal antibodies. For Bcl-xL protein expression analysis, samples of GSM cells after GSIV infection (multiplicity of infection (MOI) = 0.5) at different time points were prepared, as reported previously [23]. Briefly, after harvesting and lysis using SDS Lysis Buffer (Beyotime, Jiangsu, China), the samples were suspended in sodium dodecyl sulfate-polyacrylamide gel electrophoresis (SDS-PAGE) sample loading buffer and heated at 95 °C for 5 min. Proteins were separated by 12% SDS-PAGE and then electrotransferred to 0.22 µm polyvinylidene fluoride (PVDF) membranes using a semidry blotter (Bio-Rad, Hercules, CA, USA). The membranes were blocked subsequently in TBST (0.1% Tween-20 in Tris-buffered saline, pH 7.5) containing 5% skim milk at room temperature for 2 h. The membranes were then incubated with anti-Bcl-xL polyclonal antibodies at a dilution of 1:1000 and anti-β-actin antibody (CST, Danvers, MA, USA) at a dilution of 1:2000, separately, at 4 °C overnight. After three washes with TBST, the membranes were incubated with horseradish peroxidase (HRP)-labeled mouse-anti-rabbit IgG (CST, Danvers, MA, USA) or rabbit-anti-mouse IgG (CST, Danvers, MA, USA) and rinsed with TBST another three times. Thereafter, the membranes were incubated in a Western Lightning ECL substrate system (Perkin Elmer, Waltham, MA, USA) before exposure to a ChemiDoc™ XRS+ imaging system (Bio-Rad). β-actin was used as a reference.

For GSIV major capsid protein (MCP) expression detection, protein samples from pCDNA3.1-flag-Bcl-xL or pCDNA3.1-flag-transfected GSM cells infected with GSIV (MOI = 0.5) at different time points were prepared, as above. The protein samples were then subjected to Western blotting using an anti-MCP monoclonal antibody at a dilution of 1:1000 and an anti-β-actin antibody (both CST, Danvers, MA, USA) at a dilution of 1:2000.

For p53 expression detection, protein samples of pCDNA3.1-flag-Bcl-xL or pCDNA3.1-flag-transfected GSM cells infected with GSIV (MOI = 0.5) at different time points were prepared, as above. The protein samples were then subjected to Western blotting using an anti-p53 mouse antibody (CST, Danvers, MA, USA) at a dilution of 1:1000, and an anti-β-actin antibody (CST, Danvers, MA, USA) at a dilution of 1:2000.

### 2.6. Subcellular Localization

GSM cells were cultured in confocal microscopy wells and transfected with pDsRed-Monomer-N1-Bcl-xL and pDsRed-Monomer-N1, respectively. After infection with GSIV (MOI = 0.5) for 24 h, the cells were stained with MitoTracker^®^ mitochondrion-selective probes (Invitrogen) for 30 min at the culture temperature in the dark. The cells were then washed three times with PBS and fixed using 4% formaldehyde. The cells were stained with 4′, 6-diamidino-2-phenylindole (DAPI) (10 µg/mL) (Solarbio, Beijing, China) for 10 min at room temperature in the dark. After washing three times with PBS, the cells were observed under a confocal microscope (Olympus).

### 2.7. Flow Cytometry

GSM cells were seeded into 12-well culture plates at 20 °C for 24 h and then transfected with pCDNA3.1-flag-Bcl-xL or pCDNA3.1-flag, as stated in Section 2.4, and cells treated with Lipofectamine™ 2000 were used as a blank control. After 48 h, cells were infected with GSIV (MOI = 0.5). At 0, 24, and 48 h post infection, the cells were harvested and washed with PBS. The cells were then suspended in 210 µL staining solution containing annexin V-fluorescein isothiocyanate (FITC) and propidium iodide (PI; Beyotime) and incubated at room temperature for 20 min in the dark. After washing, the fluorescence signals were determined using a FACScan flow cytometer (Beckman Coulter, Indianapolis, IN, USA). FlowJo software (Beckman Coulter) was used for flow cytometric analysis.

### 2.8. RNA Interference

Small interfering RNAs (siRNAs) targeting AdBcl-xL (AdBcl-xL1, AdBcl-xL2, and AdBcl-xL3) were synthesized by GenePharma (Suzhou, China), and the sequences are shown in Table 1.

GSM cells were transfected with siRNAs targeting AdBcl-xL or a negative control siRNA control, and cells treated with Lipofectamine™ 2000 were used as blanks. After 48 h, cells were harvested and used for RNA extraction and cDNA synthesis. Quantitative real-time reverse transcription PCR (qRT-PCR) was used to identify the most efficient siRNA. qRT-PCR was performed in a Roter-Gene Q PCR system (Qiagen, Hilden, Germany) using a SYBR Green Dye Kit (Toyobo, Tokyo, Japan) with primers RT-F1 and RT-R1 (Table 1). The qPCR reaction was performed in a 20 µL reaction mixture containing 10 µL of 2 × SYBR Green premixture, 1 µL of diluted template cDNA (300 ng/µL), 0.5 µL of each target gene primer (10 µM), and 8 µL water. qPCR was performed using the following program: 95 °C for 30 s, then 40 cycles of 95 °C for 15 s, 60 °C for 20 s, and 72°C for 35 s, followed by an extension at 72 °C for 10 min. The mRNA levels of the genes were analyzed using the comparative threshold cycle method (2^−ΔΔCT^) [27] with β-actin as an internal reference. The GSM cells were then transfected with the plasmid encoding the most efficient siRNA-targeting AdBcl-xL and the negative control, and the cells were infected with GSIV (MOI = 0.5) 48 h post transfection. The cells were stained with Annexin V-FITC and PI, detected by FACScan flow cytometry, and analyzed using FlowJo.

### 2.9. Caspase Activity Detection

The activities of caspase-3 and caspase-9 were detected using a fluorometric protease assay kit (BioVision, Milpitas, CA, USA), following the manufacturer’s instructions. Briefly, GSM cells were cultured in 24-well plates and transfected with pCDNA3.1-flag-Bcl-xL or pCDNA3.1-flag for 24 h, then infected with GSIV at an MOI of 0.5. The infected and control GSM cells were harvested at 0, 6, 12, 24, and 48 h post infection and incubated with FITC-DEVD-FMK (for caspase-3 detection) or FITC-LEHD-FMK (for caspase-9 detection) at 20 °C for 1 h, separately. After washing three times with wash buffer, the samples were resuspended in 100 µL of wash buffer and analyzed using a fluorescence plate reader (Bio Tek, Winooski, VT, USA) at excitation (Ex)/emission (Em) wavelengths of 485 and 535 nm, respectively.

### 2.10. Detection of GSIV Major Capsid Protein (MCP) Gene Copies by Droplet Digital PCR (ddPCR)

GSM cells were cultured in 12-well plates for 24 h and then transfected with pCDNA3.1-flag-Bcl-xL or pCDNA3.1-flag. Cells treated with Lipofectamine™ 2000 were used as blank controls. After 48 h, the cells were infected with GSIV at an MOI of 0.5. At 0, 24, and 48 h post infection, supernatants containing cells were harvested, and virus DNA was isolated with Viral DNA Kit (Omega Bio-Tek) according to the manufacturer’s protocols. ddPCR was performed in a QX200™ Droplet Digital PCR™ system (Bio-Rad) using a QX200 ddPCR EvaGreen Dye Kit (Bio-Rad) with primers MCP-F/MCP-R (Table 1). The ddPCR mixture consisted of 2 μL of the diluted cDNA sample, 10 μL of 2 × QX200 ddPCR EvaGreen^®^ Supermix, 0.2 μL of each primer (10 µM), and 7.6 μL H2O. The ddPCR cycle profile included 1 cycle at 95 °C for 5 min; then 40 cycle at 95 °C for 30 s, 55 °C for 1 min, 4 °C for 5 min, and 90 °C for 5 min.

### 2.11. Co-IP and Co-Localization Assays

Cultured GSM cells were co-transfected with pCDNA3.1-flag-Bcl-xL and pCDNA3.1-Myc-HisA-Bak or pCDNA3.1-flag-Bcl-xL and pCDNA3.1-Myc-HisA for 48 h. The cells were infected with or without GSIV (MOI = 0.5) for another 24 h. Protein samples from the above cells were lysed using SDS Lysis Buffer and incubated with anti-Myc magnetic beads (MedChemExpress, Monmouth Junction, NJ, USA) for 2 h at room temperature. After separation using a magnetic frame (Bio-Rad) and discarding the supernatant, the beads were washed thoroughly with PBST (0.1% Tween-20 in phosphate buffer saline, pH 7.4) and until the OD280 in the supernatant was less than 0.05. The beads were suspended in SDS-PAGE sample loading buffer and heated at 95 °C for 5 min. After removing the beads using the magnetic frame, the supernatant was separated by SDS-PAGE, transferred to PVDF membranes, and blocked with TBST containing 5% skim milk. The membranes were incubated with mouse anti-Myc or anti-Flag antibody, washed with TBST, and incubated with HRP-labeled mouse-anti-rabbit IgG. After washing, the membranes were detected, as in Section 2.5. β-actin was used as a reference.

GSM cells were cultured in confocal microscopy wells and co-transfected with pDsRed-Monomer-N1-Bcl-xL and pEGFP-N1-Bak. After 48 h, the cells were infected with or without GSIV (MOI = 0.5) for 24 h and then fixed using 4% formaldehyde. The cells were stained with DAPI (10 µg/mL) for 10 min at room temperature in the dark. After washing with PBST, the samples were observed under the confocal microscope.

### 2.12. Quantitative Real-Time Reverse-Transcription PCR (qRT-PCR) for Molecules in the p53 Pathway

To detect the RNA transcripts of scotin, apaf-1 (encoding apoptotic protease activating factor 1), pidd (encoding p53-induced death domain protein), and igf (encoding insulin-like growth factor), pCDNA3.1-flag-Bcl-xL-transfected cells, pCDNA3.1-flag-transfected cells, or non-transfected cells were infected with GSIV and collected at 0 h, 6 h, 12 h, 24 h, and 48 h post infection. RNA extraction and cDNA synthesis were performed as above. qRT-PCR was performed using the Roter-Gene Q PCR system (Qiagen) by SYBR and a green fluorescent kit (Takara, Japan) with specific primers RT-F1 and RT-R1, RT-F2 and RT-R2, RT-F3 and RT-R3, and RT-F4 and RT-R4 (Table 1). The PCR reaction was performed in a volume of 20 µL containing 10 µL of 2 × SYBR Green premixture, 1 µL of diluted template cDNA (300 ng/µL), 0.5 µL of each target gene primer (10 µM), and 8 µL of water. qPCR was performed with the following program: 95 °C for 30 s, then 40 cycles of 95 °C for 15 s, 60 °C for 20 s, and 72 °C for 35 s, followed by an extension at 72 °C for 10 min. The mRNA levels of the genes were analyzed using the 2^−ΔΔCT^ method with β-actin as the internal reference gene. All primers used for qRT-PCR are listed in Table 1.

### 2.13. Statistical Analysis

The results were expressed as the mean ± standard deviation (SD), and statistical analyses were performed using GraphPad Prism 5 software (GraphPad Software Inc. La Jolla, CA, USA). Comparisons between groups were analyzed using one-way analysis of variance (ANOVA), statistical significance was defined as *p* < 0.05, and an extremely significant difference was defined as *p* < 0.01.

## 3. Results

### 3.1. Identifications of AdBcl-xL

**Figure 1:**
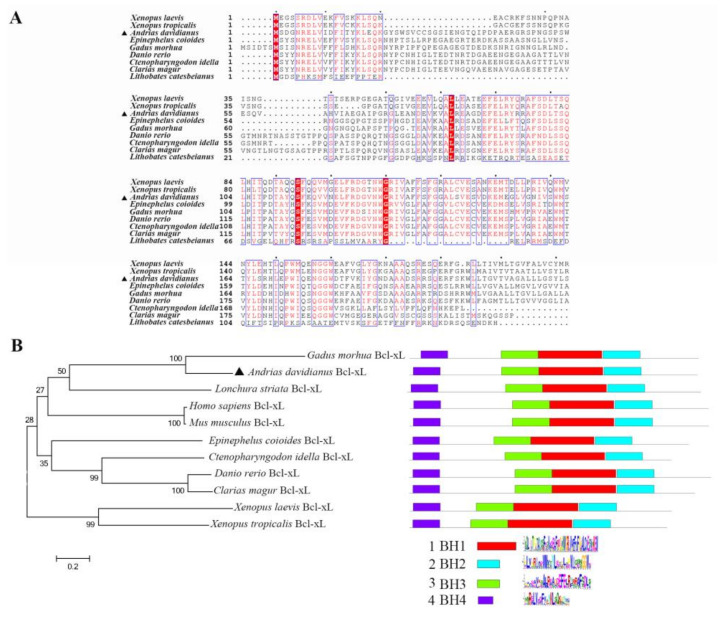
Sequence analysis of AdBcl-xL. (**A**) Alignment of the amino acid sequences of AdBcl-xL homologs in amphibians and fish. Dots denote gaps introduced for maximum matching. Numbers in brackets indicate overall sequence identities between AdBcl-xL and the compared sequences. The consensus residues are shaded in red, and residues that are ≥75% identical are identified as red characters. AdBcl-xL is marked by a triangle. (**B**) Neighbor-joining phylogenetic tree and conserved motifs of AdBcl-xL homologs from different vertebrate species. AdBcl-xL is marked by triangles. Numbers beside the internal branches indicate bootstrap values based on 1000 replications. The 0.2 scale indicates the genetic distance. Red boxes indicate BH1 domains; Blue boxes indicate BH2 domains; Green boxes indicate BH3 domains; Purple boxes indicate BH4 domains.

The ORF of AdBcl-xL encodes polypeptides of 225 amino acids (aa) (Figure S1). Sequence alignments showed that AdBcl-xL shared 52%, 51%, 49%, 48%, 47%, 44%, and 43% of overall sequence identities with the Bcl-xL homolog from amphibian and fish species, including *Xenopus laevis*, *X. tropicalis*, *Ctenopharyngodon idella*, *Clarias magur*, *Danio rerio*, *Epinephelus coioides*, and *Gadus morhua* (Figure 1). Amino acid sequence-based phylogenetic analysis showed that AdBcl-xL and Bcl-xL of G. morhua formed a cluster (Figure 1B). Similar to other Bcl-xL proteins, AdBcl-xL possesses BH1, BH2, BH3, and BH4 motifs (Figure 1B).

### 3.2. Expression and Subcellular Localization of AdBcl-xL during GSIV Infection

**Figure 2:**
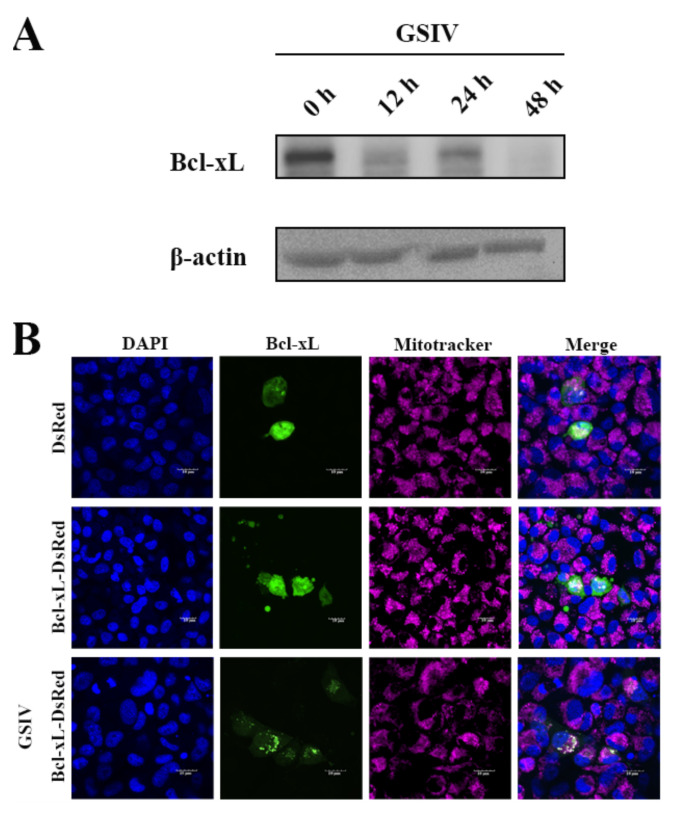
Expression and subcellular localization of AdBcl-xL after Chinese giant salamander iridovirus (GSIV) infection. (**A**) Levels of the AdBcl-xL protein at 0, 12, 24, and 48 h post GSIV infection, as assessed using Western blotting. (**B**) Cytoplasmic localization of AdBcl-xL in transfected GSM cells at 24 h post GSIV infection, as assessed using confocal microscopy. GSM cells were transfected with pDsRed-Monomer-N1-AdBcl-xL or pDsRed-Monomer-N1; mitochondria were stained with mitochondrion-selective probes; and nuclei were stained using DAPI at 48 h post transfection. Scar bar, 10 µm.

Following GSIV infection, the level of the Bcl-xL protein was reduced at 12 h post infection (hpi), increased at 24 hpi, and was finally suppressed at 48 hpi (Figure 2A). After overexpression, AdBcl-xL proteins were demonstrated to distribute irregularly in the cytoplasm of normal GSM cells, while they migrated to the mitochondria after GSIV infection (Figure 2B).

### 3.3. AdBcl-xL Negatively Regulated GSIV-Induced Apoptosis

#### 3.3.1. Overexpression of AdBcl-xL Delayed Cytopathic Effect (CPE)

**Figure 3:**
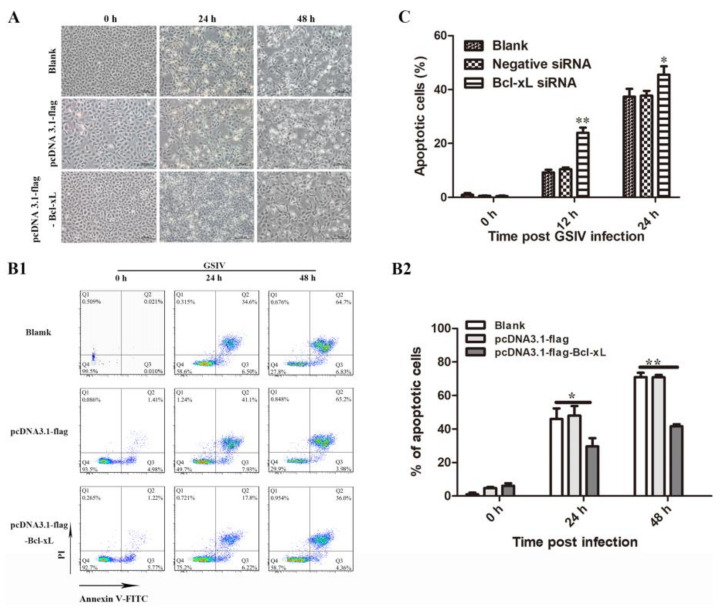
Morphological changes and apoptosis analysis of normal GSM cells, pCDNA3.1-flag transfected GSM cells and pCDNA3.1-flag-AdBcl-xL transfected GSM cells after infection with GSIV (MOI = 0.5). (**A**) Morphology and cytopathic effect (CPE) of normal GSM cells, and cells transfected with pCDNA3.1-flag or pCDNA3.1-flag-AdBcl-xL at 0, 24, and 48 h post GSIV infection. Scar bar, 100 µm. (**B1**) FACS analysis of normal GSM cells and GSM cells transfected with pCDNA3.1-flag or pCDNA3.1-flag-AdBcl-xL treated with GSIV and stained with Annexin V-FITC and PI. (**B2**) Apoptosis rate of normal GSM cells and cells transfected with pCDNA3.1-flag or pCDNA3.1-flag-AdBcl-xL during GSIV infection. (**C**) Apoptosis rate of cells transfected with the negative control siRNA, cells transfected with AdBcl-xL-siRNA and normal cells. Error bars represent the mean ± SD; * *p* < 0.05, ** *p* < 0.01. All data shown are reproducible and representative of three independent experiments.

The production of recombinant AdBcl-xL protein with the expected molecular weight in GSM cells transfected with pCDNA3.1-flag-Bcl-xL plasmid was confirmed by western blotting (Figure S2). After infected with GSIV, the cytopathic effect (CPE) in pCDNA3.1-flag-Bcl-xL transfected GSM cells appeared to be decreased compared with that in cells transfected with the empty-vector and non-transfected cells (Figure 3B).

#### 3.3.2. Overexpression of AdBcl-xL Reduced and RNA Interference of AdBcl-xL Enhanced Apoptosis Induced by GSIV

To investigate the relationship between AdBcl-xL and GSIV-induced apoptosis, GSM cells transfected with pCDNA3.1-flag-Bcl-xL or pCDNA3.1-flag and non-transfected cells, following GSIV infection, were stained with annexin v and PI and analyzed by flow cytometry (Figure 3A). The result indicated that the proportions of apoptotic cells (the sum of PI-/annexin V+ cells and PI+/annexin V+ cells) in pCDNA3.1-flag-Bcl-xL-transfected cells were significantly lower than those in pCDNA3.1-flag-transfected cells and non-transfected cells at 24 and 48 hpi (Figure 3A). In contrast, after RNA interference (RNAi), the proportions of apoptotic cells in cells transfected with the AdBcl-xL siRNA were significantly higher than those in cells transfected with the negative control siRNA and in non-transfected cells (Figure 3C).

#### 3.3.3. Overexpression of AdBcl-xL Inhibited Caspases Activity during GSIV Infection

**Figure 4:**
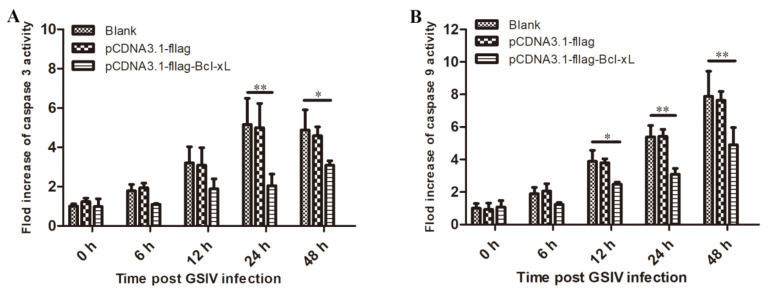
Caspase activity of normal GSM cells, pCDNA3.1-fla-transfected GSM cells, and pCDNA3.1-flag-AdBcl-xL-transfected GSM cells induced by GSIV infection at the indicated time points. Caspase-3 (**A**) and caspase-9 (**B**) activities were determined using fluorescein-active caspase-staining kits in a fluorescence plate reader. Error bars represent the mean ± SD; * *p* < 0.05, ** *p* < 0.01.

To further determine the roles of AdBcl-xL in apoptosis, the activities of caspase-3, and caspase-9 were examined. As shown in Figure 4A, caspase-9 activity in AdBcl-xL-overexpressed cells was significantly lower than that in pCDNA3.1-flag-transfected cells and in non-transfected cells at 12, 24, and 48 h after GSIV infection. Furthermore, caspase-3 activity in pCDNA3.1-Bcl-xL-transfected cells was significantly lower than that in cells transfected with the empty-vector and non-transfected cells at 12 hpi, 24 hpi, and 48 hpi (Figure 4B).

### 3.4. Overexpression of AdBcl-xL Inhibited GSIV Replication during In Vitro Infection

#### 3.4.1. Overexpression of AdBcl-xL Inhibited Copies of the GSIV MCP Gene

**Figure 5:**
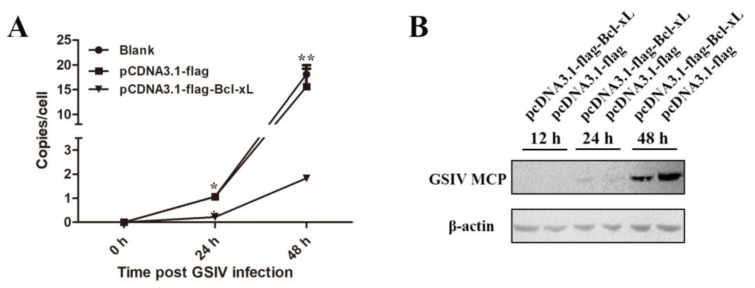
Effect of AdBcl-xL overexpression on the GSIV major capsid protein (MCP). (**A**) MCP gene copies in normal GSM cells, pCDNA3.1-flag-transfected GSM cells, or pCDNA3.1-flag-Bcl-xL-transfected GSM cells as detected using ddPCR at 6, 24, and 48 h post GSIV infection. (**B**) Proteins were extracted from GSM cells transfected with pCDNA3.1-flag-Bcl-xL and pCDNA3.1-flag at 0, 24, and 48 h post GSIV infection and analyzed by Western blotting. β-actin was used as a reference. * *p* < 0.05, ** *p* < 0.01.

In AdBcl-xL-transfected cells, the number of copies of the GSIV MCP gene was 4.4-fold and 4.6-fold lower at 24 hpi relative to that in pCDNA3.1-flag-transfected cells and non-transfected cells (Figure 5A). At 48 hpi, the number of copies of the MCP gene in AdBcl-xL-transfected cells decreased significantly by 24-fold and 21-fold, respectively, compared with that in pCDNA3.1-flag-transfected cells and non-transfected cells (Figure 5A).

#### 3.4.2. Overexpression of AdBcl-xL Inhibited GSIV MCP Protein Level

The levels of the MCP protein were analyzed using Western blotting. As shown in Figure 5B, compared with cells transfected with pCDNA3.1-flag, lower levels of MCP were detected in pCDNA3.1-flag-Bcl-xL-transfected cells at 48 hpi.

### 3.5. AdBcl-xL Directly Bound to Proapoptotic Bcl-2 Superfamily Protein AdBak

**Figure 6:**
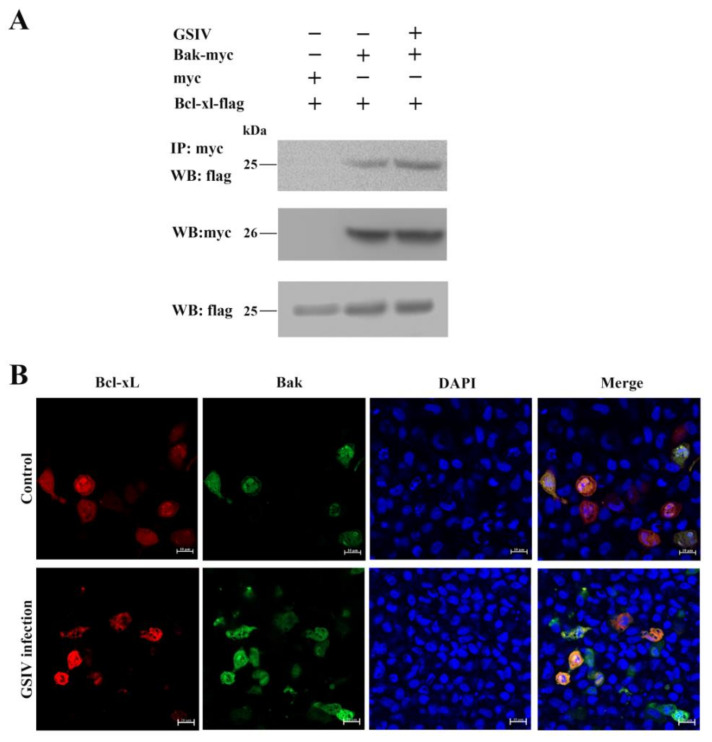
Interactions of Chinese giant salamander Bcl-xL and the proapoptotic protein Bak. (**A**) pCDNA3.1-flag-Bcl-xL and pCDNA3.1-myc-His-Bak or pCDNA3.1-myc-His were co-overexpressed in GSM cells, with or without GSIV infection, and interactions determined by coimmunoprecipitation assays using anti-Myc antibodies. (**Top**) anti-Myc pulldown, Western blotting using anti-Flag antibody; (**Middle**) Western blotting using anti-Myc antibodies; (**Bottom**) Western blotting using anti-Flag antibodies. (**B**) pDsRed-Monomer-N1-Bcl-xL and pEGFP-N1-Bak were co-overexpressed in GSM cells, with or without GSIV infection, and interactions were determined using confocal microscopy. Scale bar, 10 µm.

A co-IP assay was used to detect the interaction between the antiapoptotic molecules AdBcl-xL and the proapoptotic protein AdBak. AdBcl-xL was found to interact with AdBak (Figure 6A). Moreover, after GSIV infection, the interaction between AdBcl-xL and AdBak became stronger. These results were confirmed using confocal microscopy observation (Figure 6B).

### 3.6. Overexpression of AdBcl-xL Restained p53 Pathway

#### 3.6.1. Overexpression of AdBcl-xL Reduced p53 Level

**Figure 7:**
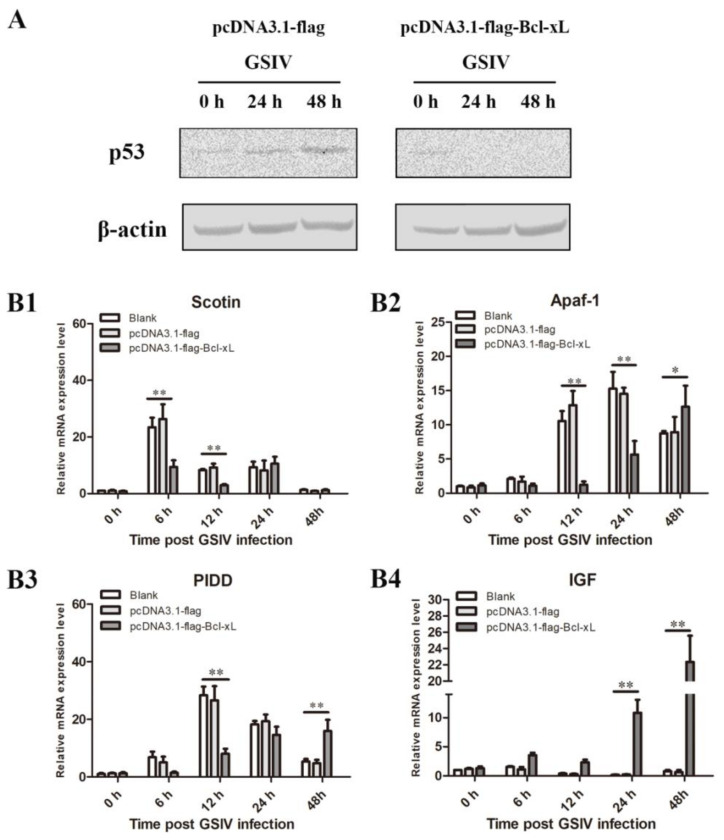
*Ad*Bcl-xL overexpression inhibited the p53 pathway during GSIV infection. (**A**) Proteins were extracted from GSM cells transfected with pCDNA3.1-flag-Bcl-xL or pCDNA3.1-flag and non-transfected cells (blank) after GSIV infection at the indicated time points and analyzed using Western blotting with β-actin as a reference. The mRNA expression patterns of p53 pathway genes, *Ad*Scotin (**B1**), *Ad*Apaf-1 (**B2**), *Ad*PIDD (**B3**), and *Ad*GF (**B4**), in GSM cells transfected with pCDNA3.1-flag-Bcl-xL or pCDNA3.1-flag and non-transfected cells (blank) were extracted after GSIV infection at the indicated time points. For convenience of comparison, the expression levels in the blank group at 0 h were set as 1. Error bars represent the mean ± SD; * *p* < 0.05, ** *p* < 0.01.

Western blotting assays showed that the level of p53 protein in AdBcl-xL-overexpressing GSM cells gradually decreased as GSIV infection progressed, while the p53 level in cells transfected with the empty vector exhibited a rising trend (Figure 7A).

#### 3.6.2. Overexpression of AdBcl-xL Regulated mRNA Expression of Molecules in p53 Pathway

The temporal expression of AdSoctin, AdApaf-1, AdPIDD, and AdIGF mRNA was examined at 0, 6, 12, 24, and 48 h post GSIV infection. Compared with those in the blank and the empty-vector groups, the transcripts of AdSoctin in AdBcl-xL-overexpressing GSM cells were significantly suppressed at 6 h (2.5-fold, *p* < 0.01) and 12 h (2.76-fold, *p* < 0.01) (Figure 7(B1)). The expression levels of AdApaf-1 in AdBcl-xL-transfected cells were significantly decreased at 12 h (8.5-fold, *p* < 0.01) and 24 h (2.7-fold, *p* < 0.01) compared with those in the blank and the empty-vector groups, followed by a slight increase in AdBcl-xL-transfected cells at 48 h (1.44-fold, *p* < 0.05) (Figure 7(B2)). The expression levels of AdPIDD in AdBcl-xL-overexpressing cells were 3.54-fold (*p* < 0.01) lower than those in the blank group and empty-vector transfected cells at 12 h, and then significantly increased at 48 h (2.98-fold, *p* < 0.01) (Figure 7(B3)). In AdBcl-xL-overexpression groups, AdIGF transcript levels increased significantly at 24 h (43.48-fold, *p* < 0.01) and 48 h (28.21-fold, *p* < 0.01) compared with those in the blank group and the empty-vector group (Figure 7(B4)).

## 4. Discussion

Bcl-xL, a BCL-2 superfamily member, contains the BCL-2 homology (BH) domains or motifs (designated as BH1, BH2, BH3, and BH4, corresponding to α-helical segments) and act as antagonists in mitochondrial apoptosis. In this study, Chinese giant salamander Bcl-xL was determined to share conserved BH1, BH2, BH3, BH4 motifs, and a C-terminal transmembrane region, which were similar to the Bcl-xL of other species. Antiapoptotic molecules in the BCL-2 superfamily are located on the MOM and control the permeabilization of the MOM by interacting with the proapoptotic molecules. We found that over-expressed Bcl-xL protein migrated to the mitochondria in the presence of GSIV. Further studies to dissect the role of AdBcl-xL in GSIV-induced apoptosis would provide new insights into the mechanism of cellular resistance against GSIV infection.

Viruses may either prevent host-cell death by expression of antiapoptotic viral proteins or promote the initiation of rapid cell death to facilitate viral progeny release following infection [28]. Studies have reported that many virus invasions lead to typical apoptotic cell death mediated by the mitochondrial pathway. As the central regulators of mitochondrial apoptosis, BCL-2 superfamily proteins are involved in this virus-induced process. In HeLa cells, overexpression of Bcl-xL blocked cytochrome c release and inhibited caspase activity during CVB3 infection [12]. Following GSIV infection, the protein level of AdBcl-xL was decreased, which implies that Bcl-xL might be a crucial component against GSIV infection. The activation of caspase-9 and caspase-3 was inhibited, and the apoptotic rate was reduced in AdBcl-xL-overexpressing cells during GSIV infection, while the apoptosis rate increased after RNAi targeting AdBcl-xL. These results indicate that Bcl-xL downregulated GSIV-induced apoptosis in GSM cells. We then investigated the impact of AdBcl-xL on virus replication. Overexpression of AdBcl-xL reduced the appearance of a CPE and reduced the number of MCP gene copies and its protein synthesis, suggesting that GSIV replication was suppressed in the presence of excess Bcl-xL. Collectively, these results demonstrate that GSIV-induced apoptosis and viral replication could be inhibited by the expression of AdBcl-xL.

Prevention of Bak oligomerization is a pivotal step toward protecting cell survival. The antiapoptotic BCL-2 proteins (e.g., Bcl-2, Bcl-xL, and MCL-1) play fundamental roles in preventing Bak oligomerization through sequestration of BH-3-only protein activators (e.g., Bid and Bim) or active Bak [29]. Upregulation at the transcriptional level of proapoptotic Bak was observed following coronavirus infection, and the onset of apoptosis was delayed in cells silenced with short interfering RNA targeting Bak, which indicates that viruses could modulate apoptosis by inducing Bak expression [30]. The results of the present study suggest that AdBcl-xL can interact directly with AdBak under GSIV infection or in normal conditions. The results indicate that Bcl-xL might inhibit GSIV-induced apoptosis by binding with Bak to preventing Bak oligomerization and thus delay apoptosis. Interestingly, after GSIV infection, AdBcl-xL and AdBak exhibited a slightly higher binding capacity. This suggests that AdBcl-xL is advantageous to prevent Bak oligomerization during GSIV-induced apoptosis. Recently, Bcl-xL was found to prevent the formation of Bak oligomers by directly restraining and retrotranslocating monomeric Bak. Which form of mitochondrial monomeric and oligomerized Bak can be retrotranslocated by Bcl-xL will be the subject of future investigation.

Researchers reported that p53 participates in the defense against viral infection by activating cell-cycle arrest or apoptosis via the transcription of target genes [31]. p53-dependent apoptosis has been identified as a powerful control to restrict virus infection. In carp, Spring viremia of carp virus (SVCV) degraded p53 early during infection, while an increase in p53 was observed late in the infection process [32]. In our previous study, the protein level of p53 was upregulated following GSIV-induced apoptosis [23]. BCL-2 proteins such as Bax/Bak are believed to be the downstream effectors of the tumor suppressor p53 [33]. In this study, overexpression of Bcl-xL blocked p53 protein expression during GSIV infection. Moreover, the mRNA expression levels of positive regulators in the p53 pathway were restrained, and the level of negative regulator was heightened following GSIV infection. Taken all together, we speculate that expression of Bcl-xL could directly bind with Bak, depress the p53 pathway, and finally inhibit GSIV-induced mitochondrial apoptosis.

Apoptosis is a crucial regulator of virus-induced cell death for their progeny release. These viruses take advantage of stimulating apoptosis, either to kill uninfected cells from the immune system or to induce the breakdown of infected cells, thereby favoring viral dissemination. Otherwise, some viruses disable host-cell apoptosis progress in the early stage of their infection since the elimination of infected cells via programmed cell death is one of the most ancestral defense mechanisms against infection [34]. The game between host and virus is a complex process, and it will be further investigated. Virus-induced apoptosis and viral mechanisms that regulate this cell-death program are key issues in understanding virus-host interactions and viral pathogenesis. Our study demonstrates that Bcl-xL can delay GSIV-induced mitochondrial apoptosis and reduce virus replication in Chinese giant salamanders. Much more efforts will be needed to clarify the exact role of Bcl-xL in regulating GSIV infection, and further research directed toward the development of selective drugs upregulating BCL-xL is still indispensable.

## 5. Conclusions

In summary, Chinese giant salamander iridovirus (GSIV) infection inhibits the expression of AdBcl-xL, and overexpression suppresses virus replication. Furthermore, AdBcl-xL inhibits the mitochondrial apoptotic cell death induced by GSIV by binding to Bak, which reduces caspase-3 and caspase-9 activation and inhibits the p53 pathway. These findings not only contribute to our understanding of the functions of the BCL-2 superfamily but also provide new insights into the complex process of iridovirus infection.

## Figures and Tables

**Table 1:** Primers used in this study.

Primer	Sequence (5′-3′)	Applications
P1	ATGGCGGAATGGAACCACCATG	PCR
P2	TCAGGGTCCAAGGAAACGGCGTA	PCR
P3	ATGGCGAACGGCGCCCC	PCR
P4	TTATTTGCTTGCAAAGAGCGCT	PCR
MCP-F	GCGGTTCTCACACGCAGTC	ddPCR
MCP-R	ACGGGAGTGACGCAGGTGT	ddPCR
AdBcl-xL1 target gene	ACAGAGAACTAGTGATTGA	RNAi
AdBcl-xL2 target gene	CTGTTCTGGTAGCATTGA	RNAi
AdBcl-xL3 target gene	GGAGATGAGTTTGAACTGA	RNAi
RT-F1	CCGTCCGTCTGCAACTCCATC	RT-PCR
RT-R1	GGGTAAATGACCAGGGCAATA	RT-PCR
RT-F2	ACAGCTATGCCTACATCTCCCTCTA	RT-PCR
RT-R2	GTTCTGGCCTGGTCACAAAGAC	RT-PCR
RT-F3	TTGGGTGAAGCTACAGCACCATG	RT-PCR
RT-R3	GCTTTCCCTTTGGCTCCTCCTT	RT-PCR
RT-F4	CGGAGACAGGCTTTTATTTCA	RT-PCR
RT-R4	AACTTCCTTTTGTGCTTTTGG	RT-PCR

## Data Availability

The data that support the fundings of this study are available from the corresponding author upon reasonable request.

## Supplementary Materials

The following are available online at https://www.mdpi.com/article/10.3390/v13112224/s1, Figure S1: cDNA sequences and deduced amino acids of AdBcl-xL, Figure S2: Expression of AdBcl-xL (A) and AdBak (B) in GSM cells by Western blot analysis, Figure S3: Efficiency detection of siRNAs targeting AdBcl-xL by qRT-PCR, Figure S4: Flow diagrams of the analysis after transfection.
