# Supplementary material for: Bcl-xL Reduces Chinese Giant Salamander Iridovirus-Induced Mitochondrial Apoptosis by Interacting with Bak and Inhibiting the p53 Pathway
Source: Viruses. 2021 Nov 4;13(11):2224. doi: 10.3390/v13112224 (PMC8622046; doi:10.3390/v13112224)

Figure S1. cDNA sequences and deduced amino acids of AdBcl-xL. The start and stop codons are underlined with full line and double line, respectively. The asterisk indicates peptide ending.

```
                   10        20        30        40        50        60        70        80        90
1      ATGTCTGATGGTAACAGAGAACTAGTGATTGACTTTATAACCTACAAGTTGGAGCAGAAGGGATACAGCTGGAGTGTTTGCTGTTCTGGT
1       M  S  D  G  N  R  E  L  V  I  D  F  I  T  Y  K  L  E  Q  K  G  Y  S  W  S  V  C  C  S  G

                  100       110       120       130       140       150       160       170       180
91     AGCATTGAAAATGGGACGCAGATCCCAGACCCAGCTGAGCGAGGTCGTTCGCCCAATGGGAGTCCTTCCTGGGAGTCCCAGGTTGCCCAT
31      S  I  E  N  G  T  Q  I  P  D  P  A  E  R  G  R  S  P  N  G  S  P  S  W  E  S  Q  V  A  H

                  190       200       210       220       230       240       250       260       270
181    GTGATAGCCGAGGGTGCCATCCCAGGCAGGGGGCTAGAAGCCAACGACGAGGTGAAGGTGGCCCTCAGAGAGGCGGGAGATGAGTTTGAA
61      V  I  A  E  G  A  I  P  G  R  G  L  E  A  N  D  E  V  K  V  A  L  R  E  A  G  D  E  F  E

                  280       290       300       310       320       330       340       350       360
271    CTGAGGTATCGCAGGGCGTTCAGCGACCTGACCTCCCAGCTGCACATCACCCCGGACACTGCGTACCAGAGCTTCGAACAGGTCGTGAAC
91      L  R  Y  R  R  A  F  S  D  L  T  S  Q  L  H  I  T  P  D  T  A  Y  Q  S  F  E  Q  V  V  N

                  370       380       390       400       410       420       430       440       450
361    GAACTCTTCCGGGACGGGGTGAATTGGGGCCGCATTGTGGCCTTCTTCTCCTTTGGCGGAGCCCTCAGCGTGGAGAGTGTCGACAAGGAG
121     E  L  F  R  D  G  V  N  W  G  R  I  V  A  F  F  S  F  G  G  A  L  S  V  E  S  V  D  K  E

                  460       470       480       490       500       510       520       530       540
451    ATGGAGGGACTCGTAGGGAACATTGTCTCATGGATGTCCACTTACCTGAGCAGGCACCTGGAGCCCTGGATCCAGGAGAACGGAGGCTGG
151     M  E  G  L  V  G  N  I  V  S  W  M  S  T  Y  L  S  R  H  L  E  P  W  I  Q  E  N  G  G  W

                  550       560       570       580       590       600       610       620       630
541    GACACGTTTGTGAAGATTTACGGCAATGATGCAGCGGCAGACAGCAGGAGGAGCCAGGAGAGGTTCAGCAAGTGGCTGCTCACCGGGGTC
181     D  T  F  V  K  I  Y  G  N  D  A  A  A  D  S  R  R  S  Q  E  R  F  S  K  W  L  L  T  G  V

                  640       650       660       670
631    ACTGTGGCCGGAGCACTTCTTCTGGGCTCCTACCTGAGTCGCAGATAG
211     T  V  A  G  A  L  L  L  G  S  Y  L  S  R  R  *
```

Figure S2. Expression of AdBcl-xL (A) and AdBak (B) in GSM cells by western blot analysis. Western blot was performed on equal amounts of protein harvested from pCDNA3.1-flag transfected GSM cells, pCDNA3.1-flag-Bcl-xL transfected GSM cells, pCDNA3.1-myc-His transfected GSM cells or pCDNA3.1-myc-His-Bak transfected GSM cells at 48 h post transfection using anti-flag monoclonal antibody.

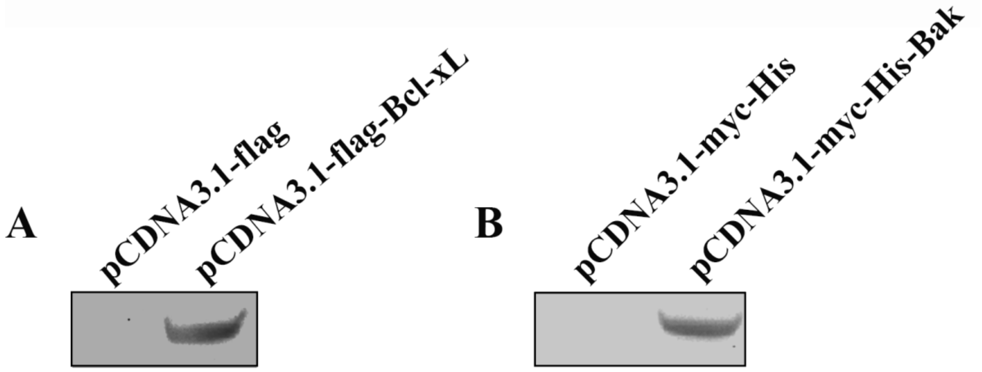

Figure S3. Efficiency detection of siRNAs targeting AdBcl-xL by qRT-PCR. Analysis of AdBcl-xL mRNA levels in GSM cells after siRNA transfection was used to confirm the RNAi knockdown. Negative siRNA transfected cells were used as control. Error bars represent as mean ± SD; ** $p < 0.01$.

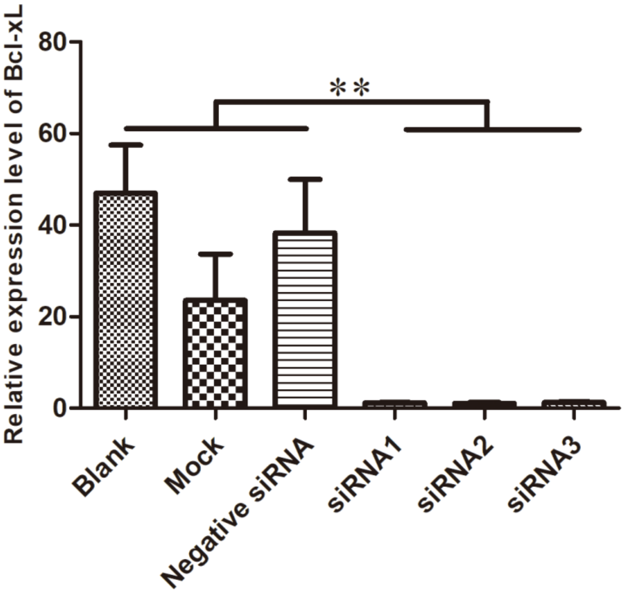

Figure S4. Flow diagrams of the analysis after transfection. A. Flow diagram of apoptosis related analysis and virus replication detection after over-expression of Bcl-xL druing GSIV infection. B. Flow diagram of interaction between AdBcl-xL and AdBak druing GSIV infection.

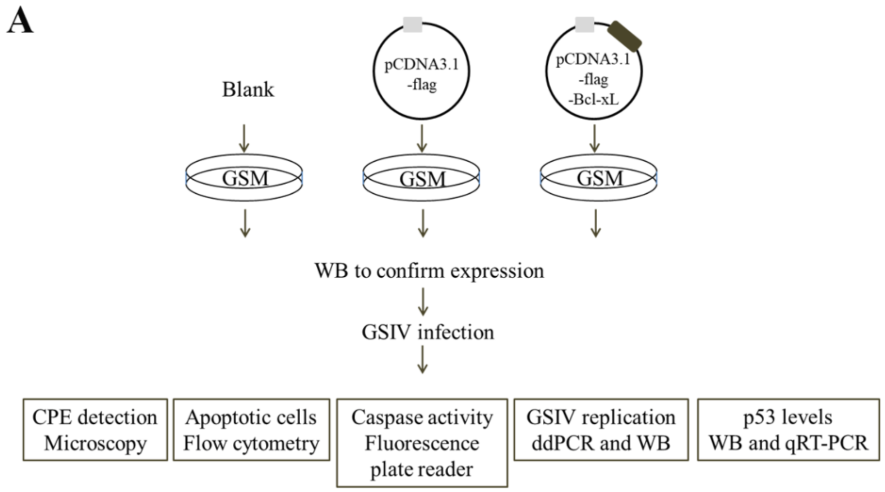

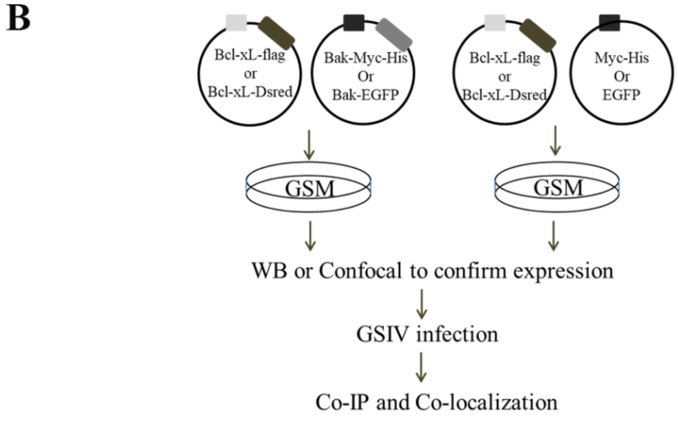

Supplement: Supplementary file 1 [file viruses-13-02224-s001.zip › viruses-1447697-supplementary.pdf]
